# Levels of Soluble NKG2D Ligands and Cancer History in Patients Starting Hemodialysis

**DOI:** 10.3389/fneph.2022.875207

**Published:** 2022-03-22

**Authors:** Kei Nagai, Takashi Tawara, Joichi Usui, Itaru Ebihara, Takashi Ishizu, Masaki Kobayashi, Yoshitaka Maeda, Hiroaki Kobayashi, Kunihiro Yamagata

**Affiliations:** ^1^ Department of Nephrology, Hitachi General Hospital, Hitachi, Japan; ^2^ Department of Nephrology, Faculty of Medicine, University of Tsukuba, Tsukuba, Japan; ^3^ Department of Nephrology, Mito Kyodo General Hospital, Mito, Japan; ^4^ Department of Nephrology, Mito Saiseikai General Hospital, Mito, Japan; ^5^ Department of Nephrology, Ushiku Aiwa Hospital, Ushiku, Japan; ^6^ Department of Nephrology, Tokyo Medical University Kasumigaura Hospital, Ami, Japan; ^7^ Department of Nephrology, JA Toride Medical Center, Toride, Japan; ^8^ Department of Nephrology, Ibaraki Prefectural Central Hospital, Kasama, Japan

**Keywords:** natural killer cell (NK cells), end-stage kidney disease (ESKD), natural killer cell receptor G2D (NKG2D), cancer, MHC class I chain-related genes A (MICA), MHC class I chain-related genes B (MICB), UL-16-binding protein (ULBP) family

## Abstract

**Background:**

Immune dysfunction in hemodialysis patients is partially due to NK cell impairment. Ligands for NK activating receptors such as NKG2D expressed on cancer cells are involved in NK cell dysfunction and can lead to cancer development.

**Methods:**

A cohort with 370 patients who started hemodialysis (HD) was investigated. Serum levels of soluble NKG2D ligands were measured. Cancer history was defined as any cancer diagnosis at induction and hospitalization and death due to cancer during 2-year follow-up.

**Results:**

Sixty-two patients with and 308 patients without a cancer history showed mostly comparable biochemical parameters and uremic status at HD induction. Soluble MICB, ULBP-1, and ULBP-2 were detected in sera from most patients starting HD rather than MICA, the most representative NKG2D ligand. Measured NKG2D ligands, except for ULBP-1, were strongly correlated with each other. Correlations between NKG2D ligands and renal function were significant but modest in patients starting HD. Cancer history did not have any impact on levels of soluble NKG2D ligands.

**Discussion:**

Even though this investigation lacked a control cohort and serial measurement of parameters, expression patterns of NKG2D ligands were comprehensively described, and the significance of cancer in patients starting HD was elucidated for the first time. Elevated levels of soluble NKG2D ligands occurred potentially due to complex mechanisms of oxidative stress, with insufficient metabolism and excretion in a uremic milieu, but they might mask the significance of elevations in serum levels of soluble NKG2DLs in patients with a cancer history.

## Introduction

Cancer is the one of the leading reasons for death in hemodialysis (HD) patients. In epidemiological studies, patients requiring replacement therapy are reported to have an increased cancer risk (1). Several lines of evidence have demonstrated that the immune system protects the host against cancer development (2–5). For instance, reduced kidney function is considered to alter the immune response, which potentially contributes to the development of cancer (6, 7). Dysfunction of natural killer (NK) cells is responsible for patients being considered immune-compromised in end-stage kidney disease (ESKD), especially HD patients (8). In the human body, it has been found that a large number of cancer cells can be produced even in the healthy state, and these cells have the potential to develop into hematopoietic and solid organ malignancies (9). Immune cells, mainly NK cells, are eradicated each time cancer cells are formed through cell-mediated immunity to tumors (2, 3). To eliminate tumor cells differentially, NK cells must recognize and distinguish healthy “self” cells from abnormal “non-self” cells *via* receptor-ligand interactions at the cell plasma membrane (8, 10). By activation signals through their interactions, NK cells become capable of recognizing and eliminating malignant cells. This mechanism is called immunosurveillance (4, 5). However, there is currently sparse evidence for molecular mechanisms linking uremic status and tumor genesis in HD patients.

Abnormal cells such as cancer cells and stressed cells express ligands for activating NK cell receptors and induce NK cytotoxic activities (11). As a representative immunoreceptor, natural killer cell receptor G2D (NKG2D), which is predominantly expressed on NK cells, activates NK cells *via* NKG2D ligands (NKG2DLs) differentially expressed on tumor cells, but not on normal cells, which implies that NKG2D plays an important role in immunosurveillance (12). This made NKG2D one of the most intensively studied immunoreceptors of the past decades (13). Animal studies of *in vivo* cancer models strongly suggest that activated NKG2D is involved in anti-cancer immune responses (4, 5). In these models, NKG2DLs expressed on tumor cells and bound to NKG2D can activate NK cells to kill tumor cells by means of perforin-based cytotoxicity (4, 5). Upon NKG2D engagement, adoptor proteins such as DNAX-activating Protein 10 are phosphorylated and recruits both the p85 subunit of the phosphatidylinisitol-3-kinase and a Grb2-Vav1 intermediating NK activation (10). Therfore, a pronouced expression of NKG2DLs renders susceptibility to NK cell cytotoxicity.

In humans, NKG2DLs comprise two members of the major histocompatibility complex (MHC)-I, including MHC class I chain-related genes A (MICA) and B (MICB) and members of the UL-16-binding protein (ULBP) family (from ULBP-1 to 6). MICA and MICB are broadly expressed by epithelial tumors, melanom and various hematopoietic malignancies and expression of mouse NKG2DL is up-regulated during tumorgenesis (11, 14–17). At the same time, MICA molecules are released from tumor cells in a soluble form (18). Elevated levels of soluble MICA (sMICA) were found in sera of patients with various hematopoietic and non-hematopoietic cancers (18, 19). Several experiments suggested that MICA is shed from the surface of tumor cells by proper metalloproteases such as “a disintegrin and metalloproteinase (ADAM)” family: ADAM 10 and ADAM17 (19, 20). These five ligand proteins were investigated in leukemia and other cancers, and they showed significantly elevated levels with a few exceptions (14, 21–23). It is also known that cancer prognosis is related to concentration of soluble NKG2DLs, which could be used as novel prognosis markers, and suggested differences in the clinical significance of individual soluble NKG2DLs (24–26).

In the uremic milieu, patients with ESKD have both an increased membrane-bound NKG2DL, MICA, on oxidative stressed monocytes and increased soluble MICA in sera compared to healthy donors, suggesting that renal dysfunction upregulates MICA (27). The enhanced expression of MICA was proven to be dependent on excessive reactive oxygen species (ROS) and uremic serum in *in vitro* analyses (27). Though changes in NKG2DL levels are likely to be involved in the immune deficiency of ESKD patients, clinical evidence to show alteration of NK cell activation affecting cancer development and prognosis in ESKD patients is still not established. To examine the clinical implications of links between the expression and functions of NK cell receptors and cancer development, the levels of various soluble NKG2DLs in serum were examined in a cohort of patients starting HD.

## Methods

### Study Protocol and Ethics

The study was performed as part of the prospective ongoing Ibaraki Dialysis Initiation Cohort Study (iDIC) project. Other details, such as the participants’ areas of residence will be reported elsewhere (Tawara T. et al, *in preparation*). Briefly, the inclusion criteria of this study were: consecutive patients starting dialysis at each participating institute from January 2013 to December 2015; and patients who needed maintenance dialysis at each participating institute. This study was conducted according to the guidelines of the Declaration of Helsinki and was granted ethics approval by the relevant institutional review board (the main institute being the University of Tsukuba Hospital, H24-116, UMIN: 000010806). Based on the written informed consent, clinical information and laboratory data were collected at study entry *via* a uniform questionnaire prepared by dialysis physicians. The data included information about age, sex, body mass index (BMI), systolic blood pressure, diastolic blood pressure, and serological testing for serum urea nitrogen, creatinine, urea acid, beta-2 microglobulin, and albumin concentration, hemoglobin, serum lipid status, and hemoglobin A1c. This cohort lacked information regarding chronic viral infections, including hepatitis B, hepatitis C, and human immunodeficiency virus. Information on death and hospitalization events with cause specificity in the subsequent two years was obtained from dialysis physicians. This investigation was limited to 370 cases who had sufficient serum samples in the original cohort.

### Sample Measurement

The concentrations of soluble NKG2DLs for which highly specific antibodies are available were comprehensively analyzed (28). Serum concentrations of soluble human MICA, MICB, ULBP-1, ULBP-2, and ULBP-3 were determined using a commercially available enzyme-linked immunosorbent assay (ELISA) kit system (R&D systems, Minneapolis, MN). ELISA was performed in accordance with the manufacturer’s protocol. Brifly, plates (Nunc-Immuno Plate; NUNC, Roskilde, Denmark) were coated with 4 μg ml^−1^ of capture antibodies in phosphate-buffered saline (PBS) overnight at 4°C, washed with 0.05% Tween 20 in PBS and blocked with 3% bovine serum albumin (BSA) in PBS before serially diluted sera samples and standard proteins were added. After conditioning, each serum sample was appropriately diluted from 1:10 to 1:1000. After 18 h of incubation at 4°C, biotinylated detection antibodies was added and incubated for 2 h at room temperature. The plates were washed, and then streptavidin–horseradish peroxidase conjugate was added for NKG2D ligands detection. After incubation for 1 h at room temperature, ABTS peroxidase substrate (506601, SeraCare Life Sciences, Milford, MA, USA) was added and absorbance was measured with a microplate reader (Bio-Rad, Hercules, CA, USA) at 405 nm. The sensitivities of the ELISAs in our laboratory were 62.5 pg/mL, 156.3 pg/mL, 93.8 pg/mL, 62.5 pg/mL, and 125.0 pg/mL, respectively, according to the data sheets. Undetected concentrations, that is those below the lower limit, were considered and analyzed as 0 ng/mL. Other biochemical parameters were evaluated as routine clinical examinations in each facility.

### Statistical Analysis

Patients with a prior history of cancer and/or death or hospitalization due to cancer during follow-up were considered to have a cancer history. Categorical variables are presented as percentages, and continuous variables are presented as means and standard deviations in **Table 1**. Contingency was assessed by the chi-squared test. A *p* value <0.05 was considered significant. Spearman correlation and partial correlation tests were performed to assess relationships between soluble NKG2DL levels and eGFR. Statistical analyses and graphical presentation were performed using SPSS version 24 and GraphPad Prism version 6, as appropriate.

**Table 1:** Study population and characterization of patients with a cancer history.

	Cancer history (-)		Cancer history (+)		*P* value
Number	308		62		
Cause of kidney disease	Number	%	Number	%	
Diabetes Mellitus	170	55.2	22	35.5	0.004
Hypertension	45	14.6	15	24.2	0.062
Chronic glomerulonephritis	52	16.9	13	21.0	0.440
Polycystic kidney disease	8	2.6	1	1.6	0.646
Rapidly progressive glomerulonephritis	8	2.6	1	1.6	0.646
Others	16	5.2	6	9.7	0.173
Undetermined	9	2.9	4	6.5	0.168
**Value item at baseline, unit**	**Mean, S.D.**				
Age of induction, y	66	13	74	9	0.001>
Body mass index, kg/m^2^	24.5	4.8	24.4	3.8	0.877
Systolic blood pressure, mmHg	152	28	142	23	0.008
Diastolic blood pressure, mmHg	80	17	72	17	0.008
Urea Nitrogen at baseline, mg/dL	93.2	29.2	94.5	34.9	0.757
Creatinine at baseline, mg/dL	9.33	3.05	8.87	2.76	0.272
Estimated GFR, ml/min/1.73 m^2^	5.2	1.8	5.5	1.6	0.223
Urea acid, mg/dL	8.8	12.0	7.9	2.2	0.556
Beta-2 macroglobulin, mg/dL	24.9	39.5	19.4	4.5	0.274
Serum albumin, g/dL	3.7	3.6	3.3	0.6	0.384
Hemoglobin, mg/dL	9.0	2.1	8.8	1.6	0.478
Triglycerides, mg/dL	116	62	107	56	0.290
HDL-C, mg/dL	49.3	20.4	45.9	14.8	0.213
LDL-C, mg/dL	91	37	86	36	0.331
HbA1c, %	5.9	0.9	5.8	0.7	0.409

GFR, glomerular filtration rate; HDL-C, high-density lipoprotein cholesterol; LDL-C, low-density lipoprotein cholesterol; HbA1c, hemoglobin A1c; S.D., standard deviation.

## Results

**Table 1** presents the background data of the patients with and without a cancer history. The mean age of the 62 patients with a cancer history, including 54 patients with known cancer at induction and 14 hospitalizations and 8 deaths (composed of 3 cases with urethral cancer, and one case each of pancreatic cancer, rectal cancer, multiple myeloma, breast cancer, and unknown origin cancer) by including duplications, was significantly higher than that of the 308 patients without a cancer history. With respect to the causes of the patients’ renal diseases, diabetes mellitus was more common in patients without a cancer history, and higher age was more common in those with a cancer history. The two groups showed mostly comparable biochemical parameters and uremic status at dialysis induction.

**Figure 1:**
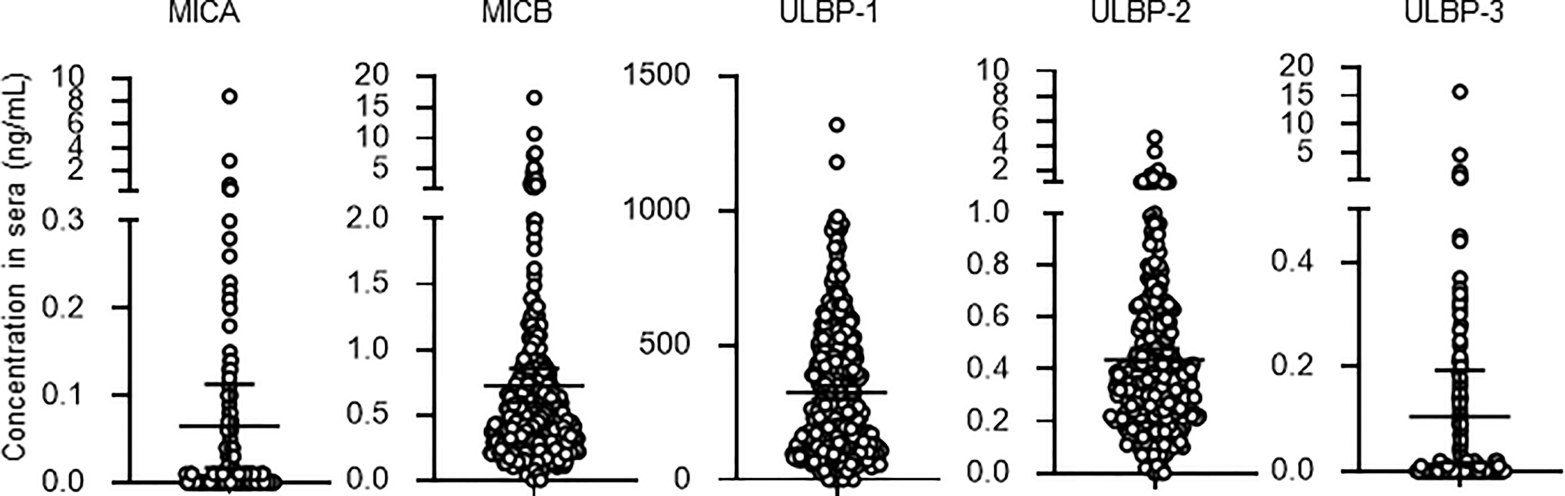
Serum concentrations of soluble NKG2D ligands in incident hemodialysis patients. Serum levels of soluble forms of major histocompatibility complex (MHC)-I including MHC class I chain-related genes A (MICA) and B (MICB) and members of the UL-16-binding protein (ULBP) family from 370 patients of the study cohort. Undetected concentrations, that this those that were below the lower limit, were considered and analyzed as 0 mg/dL. Median and 95% confidence intervals are presented as bars and error values.

**Figure 2:**
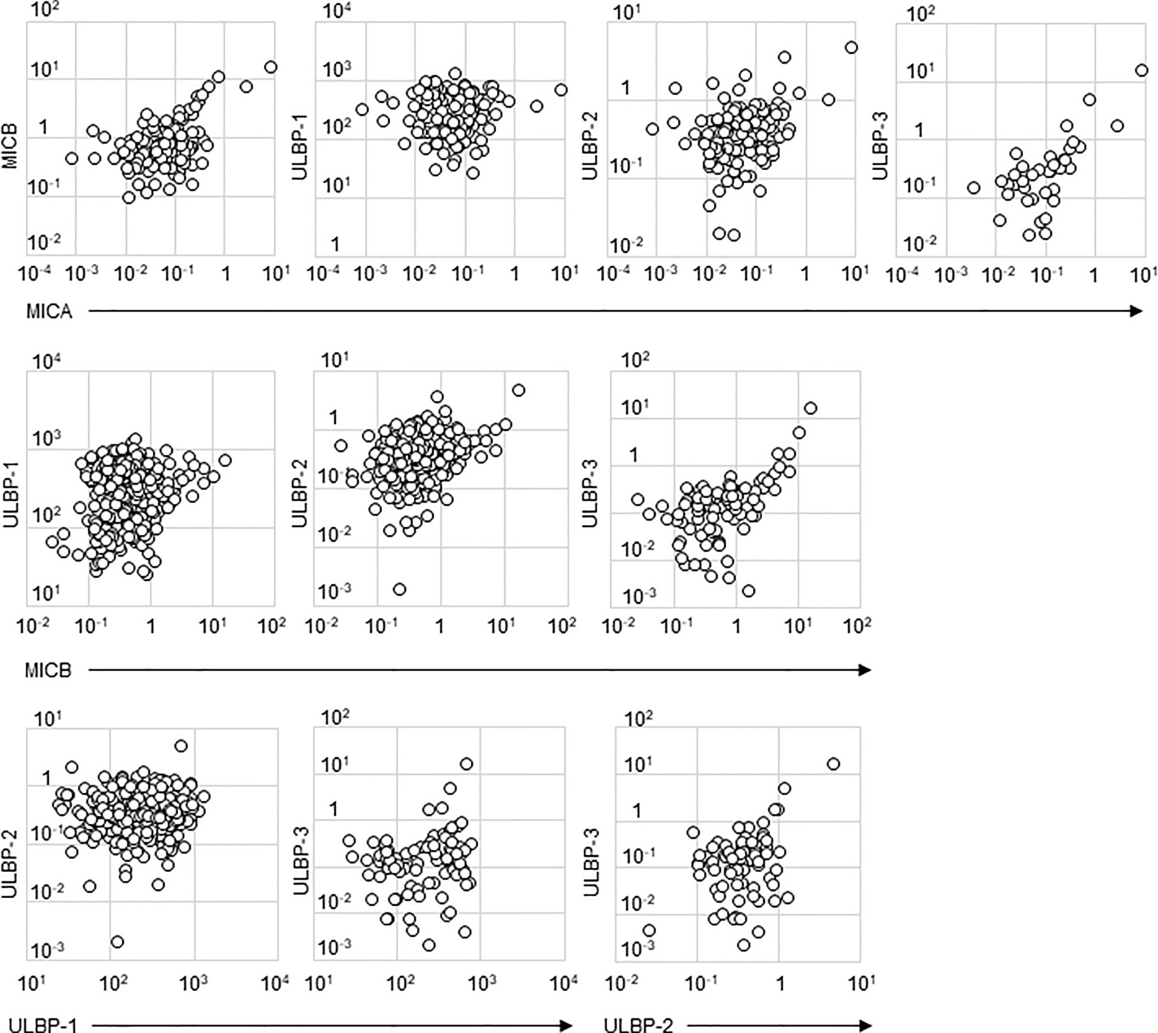
Scatter plots of soluble NKG2D ligand levels in incident hemodialysis patients. Serum levels of two types of NKG2D ligands investigated in this study are plotted in each figure panel. Logarithms are used for the axes. All the concentrations of NKG2D were shown as ng/mL. Their correlation was assessed and the results were shown in **Table 2**. major histocompatibility complex (MHC)-I including MHC class I chain-related gene A (MICA) and B (MICB); the UL-16-binding protein (ULBP) family (ULBP-1, 2 and 3).

**Figure 3:**
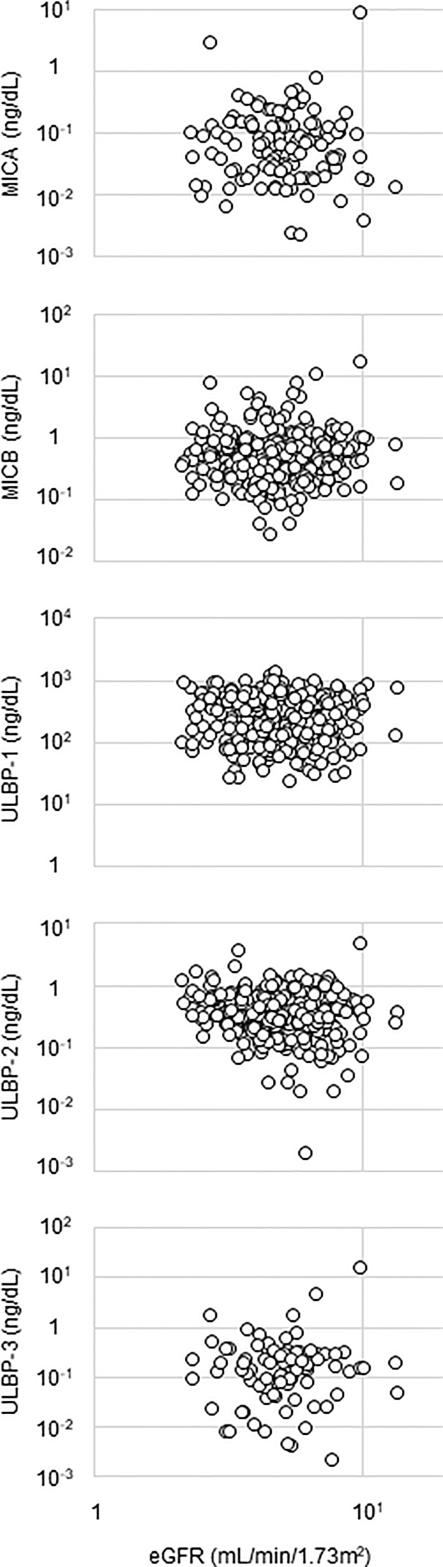
Relationship between NKG2D ligands and renal function. Serum level of a NKG2D ligand and estimated glomerular filtration ratio (eGFR) are plotted in each figure panel. Logarithms are used for the axes. Their correlation was assessed and the results were shown in **Table 2**. major histocompatibility complex (MHC)-I including MHC class I chain-related gene A (MICA) and B (MICB); the UL-16-binding protein (ULBP) family (ULBP-1, 2 and 3).

Detectable sample numbers of MICA, MICB, ULBP-1, ULBP-2, and ULBP-3 were 130, 368, 365, 368, and 86, respectively (**Figure 1**). Of the NKG2D ligands in this study cohort, MICA, MICB, ULBP-2, and ULBP-3, but not ULBP-1, were strongly correlated each other (**Table 2** and **Figure 2**), and age-adjustment by partial correlation testing did not affect the relationships (**Table 2**). **Figure 3** shows that the correlations between NKG2D ligands and residual renal function were significant, but extremely modest in patients starting HD (coefficients on age-adjusted partial correlation tests: MICA 0.103, ULBP-1 0.130).

**Table 2:** Correlations among NKG2D ligands and renal function in incident HD patients.

Bivariate		Correlation		Partial correlation (age adjustment)	
		Coefficient	P value	Coefficient	P value
MICA	MICB	0.783	<0.001**	0.788	<0.001**
MICA	ULBP-1	0.086	0.097	0.088	0.091
MICA	ULBP-2	0.575	<0.001**	0.575	<0.001**
MICA	ULBP-3	0.944	<0.001**	0.944	<0.001**
MICB	ULBP-1	0.136	0.009**	0.132	0.011*
MICB	ULBP-2	0.491	<0.001**	0.495	<0.001**
MICB	ULBP-3	0.817	<0.001**	0.820	<0.001**
ULBP-1	ULBP-2	0.052	0.317	0.053	0.306
ULBP-1	ULBP-3	0.082	0.114	0.082	0.115
ULBP-2	ULBP-3	0.562	<0.001**	0.562	<0.001**
eGFR	MICA	0.103	0.048*	0.101	0.053
eGFR	MICB	0.075	0.147	0.113	0.030*
eGFR	ULBP-1	-0.050	0.333	-0.031	0.554
eGFR	ULBP-2	-0.029	0.576	-0.038	0.462
eGFR	ULBP-3	0.130	0.012*	0.141	0.007*

major histocompatibility complex (MHC)-I including MHC class I chain-related gene A (MICA) and B (MICB); the UL-16-binding protein (ULBP) family (ULBP-1, 2 and 3); eGFR, estimated glomerular filtration rate.

*P < 0.05, **P < 0.01.

**Figure 4:**
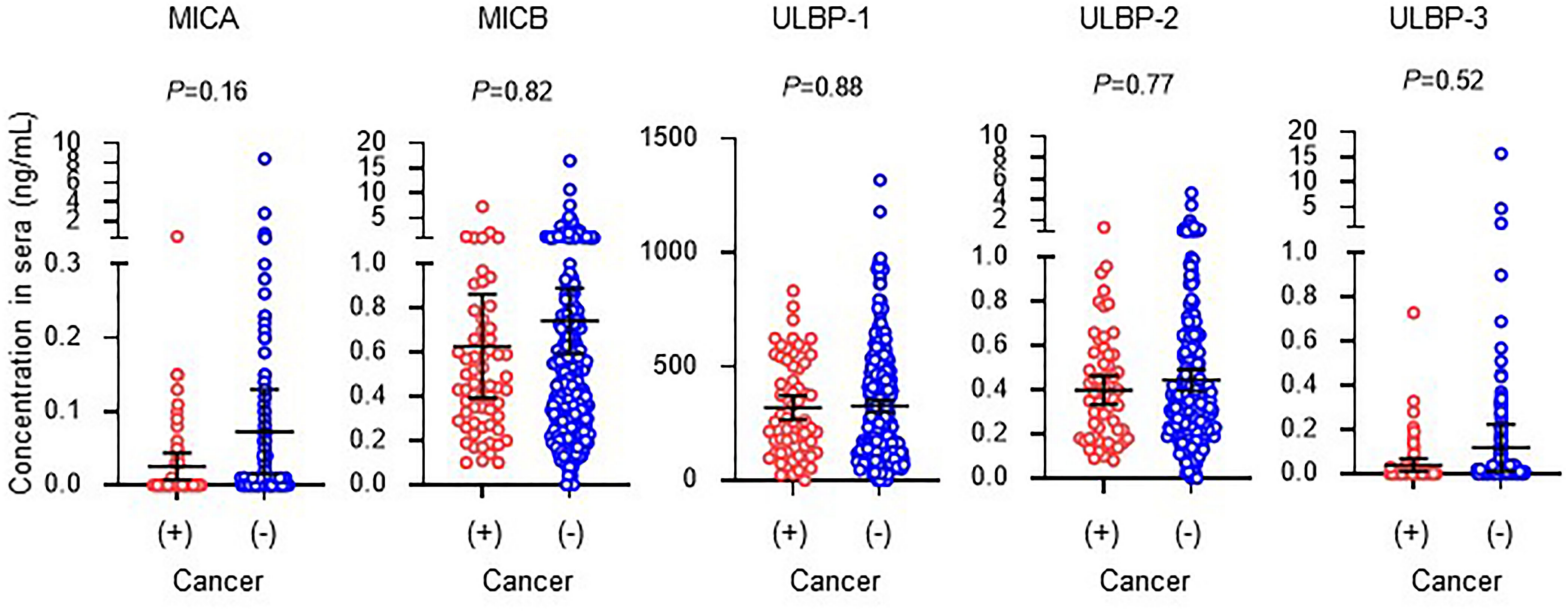
Serum concentrations of soluble NKG2D ligands in incident hemodialysis patients with or without a cancer history. Serum levels of soluble forms of major histocompatibility complex (MHC)-I including MHC class I chain-related genes A (MICA) and B (MICB) and members of the UL-16-binding protein (ULBP) family. Undetected concentrations, that this those that were below the lower limit, were considered and analyzed as 0 mg/dL. Patients with a prior history of cancer and death or hospitalization due to cancer during follow-up were considered to have a cancer history. Median and 95% confidence intervals are presented as bars and error values. Differences in median values between with and without a cancer history are compared by the Mann-Whitney *U* test.

Mean serum levels (95% confidence intervals) of soluble NKG2DLs in 308 incident HD patients without a cancer history were 0.072 (0.015-0.130) ng/mL, 0.742 (0.593-0.891) ng/mL, 327 (300-342) ng/mL, 0.444 (0.396-0491) ng/mL, and 0.118 (0.012-0.224) ng/mL, respectively in MICA, MICB, ULBP-1, ULBP-2, and ULBP-3. Those in 62 incident HD patients with a cancer history were 0.026 (0.008-0.044) ng/mL, 0.628 (0.392-0.863) ng/mL, 319 (265-373) ng/mL, 0.398 (0.333-0.464) ng/mL, and 0.039 (0.010-0.067) ng/mL, respectively in MICA, MICB, ULBP-1, ULBP-2, and ULBP-3 (**Figure 4**). Collectively, there were no differences in levels of soluble NKG2D ligands between the presence and absence of a cancer history in this study cohort with two-year follow-up (**Figure 4**).

## Discussion

In this study, soluble NKG2DL concentrations were examined in sera from patients starting HD. Mechanisms of production of soluble NKG2DLs are generally considered to be the regulation of their release by various processes, including protease-mediated cleavage (29). Particularly in the uremic state, Peraldi et al. characterized NKG2D and MICA in healthy control and uremic patients, and they concluded that oxidative stress-related NKG2D downregulation and up-regulation of MICA protein may be involved in the immune deficiency of ESKD patients (27). Though this study sufficiently demonstrated biological dysfunction of NK cells in HD patients, little supporting clinical implication was reported. Serum levels of NKG2DLs vary with regard to regulation of their expressions, surface attachment, and affinities to NKG2D, suggesting differential roles in NK cell biology (21). The axis of NKG2D and its ligands had been well investigated and most established in the context of cancer immunosurveillance (21). In particular, soluble MICA levels, but also other soluble NKG2DLs, have been analyzed in a multitude of different malignancies (22). For example, in one large-sized study (205 leukemia patients and 30 healthy controls), there were significantly elevated levels of soluble MICA, MICB, ULBP-1, ULBP-2 and ULBP-3 in all leukemia entities except soluble ULBP-2 in chronic myeloid leukemia (CML), soluble ULBP-3 in chronic lymphocytic leukemia (CLL), and soluble ULBP-1 in acute myeloid leukemia (AML) (28). This suggests that the differences in the functional properties of NKG2DLs may provide an explanation as to why the various NKG2DLs differ in their correlations with diseases. Therefore, expressions of five NKG2DLs were comprehensively studied in 370 patients starting HD, and the clinical impact on cancer history, namely, past history of cancer, and/or hospitalization and death due to cancer was examined in two-year follow-up.

One of the main findings of the present study was that, rather than MICA, soluble MICB, ULBP-1, and ULBP-2 were detected in sera from most patients starting HD. Levels of sMICA in healthy donors were undetectable or in the very low value range, whereas pre-therapeutic serum levels in patients with various malignancies were significantly higher (30). Regarding soluble NKG2DLs other than sMICA, neither sULBP-1 nor sULBP-3 levels were detectable in the blood samples of healthy controls. Similarly, much lower levels of sMICB and sULBP-2 were seen in healthy controls compared to CLL patients (31). Though the present study cohort did not contain healthy controls or patients with normal renal function, one can assume that detectable levels of NKG2DLs are likely to occur due to renal dysfunction. In addition to oxidative stress in the uremic milieu, insufficient metabolism and excretion should be considered as causes of elevated levels of NKG2DLs in incident HD patients (32). In this study, eGFR was significantly but very modestly correlated only with MICB and ULBP-3. Thus, one cannot conclude whether reduced renal function is linked with detectable levels of serum sNKG2DLs, possibly due to the narrow range of eGFR in the study population (eGFR 5.3 ± 1.8, ranging from 2.1 to 13.7 ml/min/1.73 m^2^).

A pathological rationale for the soluble form is to down-modulate cell surface expression of ligand protein on tumor cells. In addition, soluble MICA can cause the downregulation of surface expression of NKG2D receptors by promoting their internalization and degradation, leading to reduced immune responses against tumors (33). These pathways contribute to escape from immune surveillance by host NK cell immunity and elevation in soluble NKG2DLs considered to result in progression of malignant disease and metastasis *in vivo*. Therefore, soluble NKG2DLs levels are expected to be markers of tumor progression and prognosis, because soluble MICA and ULBP-2 levels distinguish significantly between benign and malignant disease, both in solid organ cancers and in hematopoietic cancers (22, 31). However, based on the present investigation, these soluble NKG2DLs seemed to fail to distinguish patients with a cancer history in the incident HD cohort. This might be mainly caused by the effect of renal dysfunction and excessive oxidative stress on masking elevation of sNKG2DLs produced by tumor cells in HD patients. However, further examinations are needed to characterize the links between renal dysfunction and elevations in soluble NKG2DL levels in sera of HD patients.

Most previous laboratory investigations did not provide any clinical implications in ESKD patients. In fact, compared to previous studies, a major drawback the present investigation is the lack of a control group and lack of cell surface expression analyses of NKG2DLs. Nevertheless, the present research has novelty from the perspective of exploring clinical significance in an incident HD cohort, though no difference was seen between the presence and absence of a cancer history. Recently, Dendle et al. reported that reduced NK cytotoxic activity assessed *in vitro* is associated with NK cell-related infectious complications in ESKD (34). We first assessed cancer, another NK cell-related clinical complication (35, 36), as a clinical outcome in an ESKD cohort. Further clinical research on the relationship between NK cell function and development of cancer in dialysis patients is desirable.

As limitations, in this cohort study, biomarkers were only available at baseline at the time of starting HD. Therefore, changes in levels of soluble NKG2DLs could not be examined, so it is not possible to determine whether there was new onset of cancer during follow-up or there was pre-existing cancer at baseline. Another problem is the absence of a control group; therefore, correlations between parameters and soluble NKG2D levels were examined at baseline only in a cross-sectional manner. Because a cancer history was determined based on a uniform questionnaire prepared by physicians, reporting bias is considerable. Moreover, this cohort had no information regarding chronic viral infections, which may have an effect on expression levels of NKG2D and NKG2DLs and may mask the significance of elevations in serum levels of soluble NKG2DLs in patients with a cancer history.

In summary, serum levels of soluble NKG2DLs were examined in 370 incident HD patients to explore the clinical implications of links between expression of NK cell receptor function and cancer development. MICB, ULBP-1, and ULBP-2, but not MICA and ULBP-3, were detected in sera from most patients starting HD, and it was difficult to discriminate between the presence and absence of a cancer history using the levels of soluble NKG2DLs. This difficulty may have occurred potentially due to the complex mechanisms of oxidative stress, insufficient metabolism, and excretion in the uremic milieu.

## Data Availability Statement

The data analyzed in this study is subject to the following licenses/restrictions: The data that support the findings of this study are available on request to steering committee of iDIC study. Requests to access these datasets should be directed to KY, https://nephtsukuba.wixsite.com/nephrology-tsukuba/idic.

## Ethics Statement

The studies involving human participants were reviewed and approved by University of Tsukuba Hospital. The patients/participants provided their written informed consent to participate in this study.

## Author Contributions

The corresponding author (KN) designed this research and was involved in measurements of samples and data analyses with supervision by JU and TT. The chief investigators of the iDIC study (KY), the officer of the research center (JU), and investigators (IE, TI, MK, YM, and HK) comprised the study’s steering committee. Steering committee members contributed to patient entries, design of the study, data analysis, and editing of the manuscript. All authors contributed to the article and approved the submitted version.

## Funding

This article was supported, in part, by JSPS Grant No. 18KK0431, 19K17729 and by the Japanese Association of Dialysis Physicians Grant No. 2020-3.

## Conflict of Interest

The authors declare that the research was conducted in the absence of any commercial or financial relationships that could be construed as a potential conflict of interest.

## Publisher’s Note

All claims expressed in this article are solely those of the authors and do not necessarily represent those of their affiliated organizations, or those of the publisher, the editors and the reviewers. Any product that may be evaluated in this article, or claim that may be made by its manufacturer, is not guaranteed or endorsed by the publisher.
